# Using stable isotopes to infer stock‐specific high‐seas distribution of maturing sockeye salmon in the North Pacific

**DOI:** 10.1002/ece3.7022

**Published:** 2020-11-13

**Authors:** Boris Espinasse, Brian P. V. Hunt, Bruce P. Finney, Jeffrey K. Fryer, Alexander V. Bugaev, Evgeny A. Pakhomov

**Affiliations:** ^1^ Department of Earth, Ocean and Atmospheric Sciences University of British Columbia Vancouver British Columbia Canada; ^2^ Institute for the Oceans and Fisheries University of British Columbia AERL Vancouver BC Canada; ^3^ Hakai Institute Heriot bay British Columbia Canada; ^4^ Department of Biological Sciences Idaho State University Pocatello ID USA; ^5^ Department of Geosciences Idaho State University Pocatello ID USA; ^6^ Columbia Inter‐Tribal Fish Commission Portland OR USA; ^7^ Kamchatka Branch of Russian Federal Research Institute of Fisheries and Oceanography Petropavlovsk‐Kamchatsky Russia; ^8^ Present address: Arctic and Marine System Ecology Faculty of Biosciences, Fisheries and Economy UiT The Arctic University of Norway Tromsø Norway

**Keywords:** animal tracking, biogeography, feeding grounds, scales, δ^13^C and δ^15^N

## Abstract

The stock‐specific distribution of maturing salmon in the North Pacific has been a persistent information gap that has prevented us from determining the ocean conditions experienced by individual stocks. This continues to impede understanding of the role of ocean conditions in stock‐specific population dynamics. We assessed scale archives for 17 sockeye salmon (*Oncorhynchus nerka*) stocks covering the entire North Pacific, from the Columbia River (Washington State and British Columbia) to Kamchatka Peninsula (Russia), to infer salmon locations during their last growing season before returning to their spawning grounds. The approach used, first pioneered in salmon stocks in the Atlantic, relies on the relationship between temporal changes in δ^13^C in salmon scales and sea surface temperature to estimate salmon distribution based on correlation strength. An advantage of this approach is that it does not require fish sampling at sea, but relies on existing fishery agency collections of salmon scales. Significant correlations were found for 7 of the stocks allowing us to propose plausible feeding grounds. Complementary information from δ^15^N, historical tagging studies, and connectivity analysis were used to further refine distribution estimates. This study is a first step toward estimating stock‐specific distributions of salmon in the North Pacific and provides a basis for the application of the approach to other salmon scale archives. This information has the potential to improve our ability to relate stock dynamics to ocean conditions, ultimately enabling improved stock management. For example, our estimated distributions of Bristol Bay and NE Pacific stocks demonstrated that they occupy different areas with a number of the former being distributed in the high productivity shelf waters of the Aleutian Islands and Bering Sea. This may explain why these stocks seem to have responded differently to changes in ocean conditions, and the long‐term trend of increased productivity of Bristol Bay sockeye.

## Graphical abstract

Stock‐specific salmon high seas distribution is largely unknown. We used the correlation between sea temperature and stable isotope ratio in scales of 17 salmon stocks to estimate their distributions during their last year at sea. This has resulted in a better comprehension of salmon migration routes, differences in stock productivity trends over large areas (e.g., Bristol Bay vs Northeast Pacific) and differences in productivity of stocks with geographically close spawning grounds.

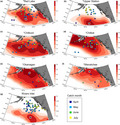

## 1 INTRODUCTION

Sockeye salmon (*Oncorhynchus nerka*) migrate from freshwater to the coastal ocean as juveniles and later in the same year migrate from the coast to the open ocean where they typically reside for 2–3 years before returning to their natal streams to spawn (Burgner, 1991). Although it is considered that the early marine life phase is the period most critical to survival, there are indications that much of the cumulative juvenile‐to‐adult mortality of salmon occurs on the high seas (McKinnell et al., 2014; Welch et al., 2011). It is also during this period, and especially during maturation, that the fish accumulate most of their mass (Ishida et al., 1998) and establish the body condition necessary to undertake successful spawning migration and reproduction. However, our understanding of the high‐seas life phase and its role on determining stock‐specific recruitment dynamics remains limited. This knowledge gap reflects the challenges posed by the massive spatial scale of the North Pacific, the wide dispersal of fish, and the associated expense and logistical challenges of sampling.

Many sockeye salmon spawning grounds are located at considerable distance from the sea (up to 100’s km), requiring substantial reserves for upstream migration. The energy requirements depend on water temperature, that will affect salmon metabolic rate, distance, and river flow strength, both of which determine the relative migration duration (Rand et al., 2006; Crossin et al., 2008; Macdonald et al., 2010). Poor body condition (smaller size, low lipid content) associated with, for example, high river discharge can result in large en‐route mortality (Macdonald, 2000). Warmer than usual temperatures experienced by the fish in the high seas is one of the factors that has been suggested to explain low salmon fitness by altering salmon phenology (early maturation) and reducing nutritional health (low lipid content) (McKinnell, 2000, 2013).

Another direct effect of a warming North Pacific Ocean on salmon is a reduction in the area of suitable thermal habitat (Abdul‐Aziz et al., 2011; Healey, 2011; Welch et al., 1998). This is expected to result in an intensification of competition for resources. Recent studies indicate that competition for food among salmon species in the open ocean can play a role in regulating populations (Ruggerone & Connors, 2015; Springer & van Vliet, 2014). Increasing pink salmon abundance (*Oncorhynchus gorbuscha*), in part due to hatchery enhancement, combined with the limited carrying capacity of the North Pacific Ocean could result in unfavorable conditions for sockeye development. However, the influence of food composition and abundance on salmon development and condition remains largely unknown, although Pacific salmon seem to be able to adapt their diet when food conditions change (Kaeriyama et al., 2004).

A prerequisite to determining the extent to which these different processes affect sockeye salmon stocks is knowledge of salmon distributions in the high seas (Chittenden et al., 2009). The state of the knowledge of maturing sockeye salmon distribution has been reviewed on a few occasions over the past decades (Burgner, 1991; Myers et al., 2007; Farley et al., 2018) and is mainly based on tagging studies carried out aboard fishery vessels (French et al., 1976). In particular, a large effort was conducted in the 1960s by the International North Pacific Fisheries Commission (INPFC) to provide first insights into stock‐specific salmon distribution, based primarily on fish caught between May and July. In the future, other approaches, such as genetic stock identification (Habicht et al., 2010) and scale pattern analysis (Bugaev et al., 2009) might contribute further distribution data. However, all of these approaches rely on collecting salmon in the open ocean, an extremely challenging task due to the enormous technical and logistical difficulty associated with sampling at ocean basin scales. As a consequence, there are still many uncertainties associated with high‐seas salmon distributions (Myers et al. 2007).

The implementation of approaches to infer salmon distribution from biogeochemical information stored in the animal tissues offers a complementary and valuable step forward to reduce these knowledge gaps. In this regard, the stable isotope ratios of carbon and nitrogen in salmon scales (expressed as δ^13^C and δ^15^N values) have proven to be a useful tool to estimate the environmental conditions experienced by fish and their distribution patterns (MacKenzie et al., 2012, 2012; Torniainen et al., 2014; Trueman et al., 2012). Animal tissue δ^13^C has been demonstrated to be a particularly reliable indicator to track animal location due to its covariance with SST (MacKenzie et al., 2012, 2012; McMahon et al., 2000, 2013; Almodóvar et al., 2020). This relationship occurs because water temperature is a dominant driver of aqueous [CO_2_], which in turns controls carbon isotope fractionation by autotrophs. Because autotrophs preferentially take up the lighter carbon isotope (^12^C), high [CO_2_] concentrations (low SST) result in low δ^13^C, and conversely a decrease in [CO_2_] concentrations (high SST) leads to higher δ^13^C as less of the lighter isotope is available for autotrophs (Lourey et al., 2004; Rau et al., 1989). Although other mechanisms linked to phytoplankton physiology and community composition do affect phytoplankton δ^13^C values (Burkhardt et al., 1999; Riebesell et al., 2000), water temperature is considered to be the main driver of δ^13^C variation at high latitude (Magozzi et al., 2017). Using this approach implies two large assumptions: (a) that stock‐specific feeding grounds have remained constant throughout the time series being considered (i.e., there is no plasticity in feeding location) and (b) that diet composition or quality (e.g., lipid content) has not altered systematically across space or over time. Recently, the δ^13^C/SST correlation has been used to identify Atlantic salmon (*Salmo salar*) feeding grounds in the North Atlantic (MacKenzie et al., 2012, 2012; Soto et al., 2018) and for one sockeye stock in the North Pacific (Espinasse et al., 2018). This approach presents the advantages of being applicable retrospectively using scale archives and does not require sampling fish at sea.

In this study, we build on the preliminary work of Espinasse et al. (2018) to assess stock‐specific sockeye salmon distributions in the North Pacific through analysis of archived scales for 17 North Pacific sockeye salmon stocks. Our primary objectives were to determine whether the δ^13^C/SST relationship could be used to provide reasonable feeding grounds estimates for the stocks studied and to explore the limits of the method in relation to the time series characteristics (e.g., length, resolution).

## 2 MATERIALS AND METHODS

### 2.1 Data collection and scale processing

**Figure 1:**
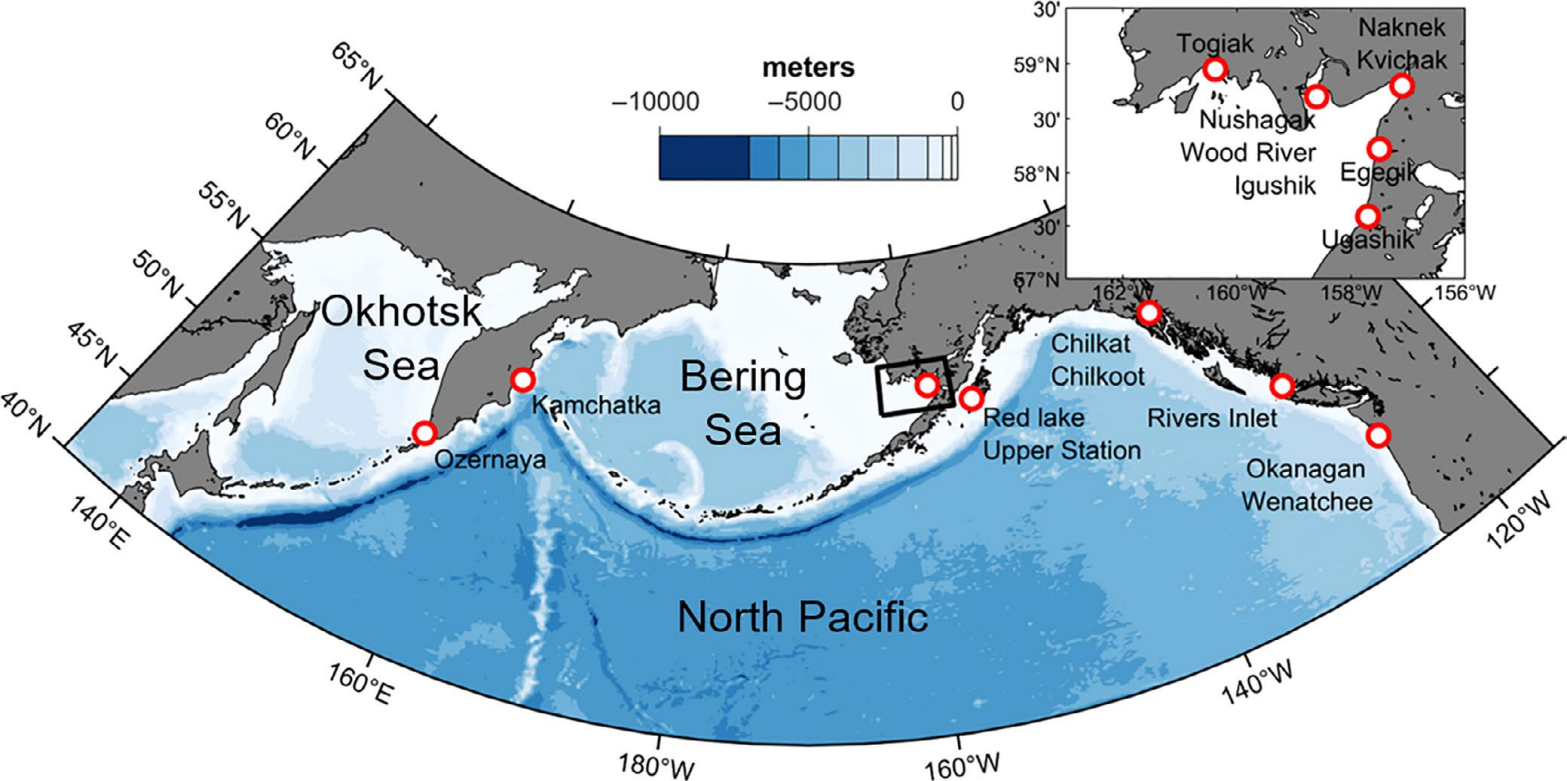
Map of the study area with coastal locations (indicated by red rings; map insert shows locations of Bristol Bay stocks) of the sockeye salmon stocks investigated. Bathymetric data are also shown (scale of blues)

The literature was reviewed for published stable isotope data of sockeye salmon scales. Johnson and Schindler (2012) reported on stable isotope data for eight stocks distributed in Bristol Bay with time series spanning over four decades of scales collected every 3 years. Satterfield and Finney (2002) reported on a 30‐year time series with yearly resolution for a stock located on Kodiak Island in SW Alaska, and Espinasse et al. (2018) reported on stable isotope data for the Rivers Inlet stock (BC coast) which covered more than 50 years with irregular sampling resolution. In addition, we accessed archived scales for two major stocks in the Kamchatka Peninsula (Ozernaya and Kamchatka) (Bugaev et al., 2008), for two stocks of the Columbia River (Okanagan and Wenatchee), for two stocks in SE Alaska (Chilkoot and Chilkat) and for one additional stock on Kodiak Island. The stock locations can be seen in Figure 1, and the details of the time series resolution are provided in Table 1.

**Table 1:** Details of the salmon scale samples analyzed (*n* = 1,995), δ^13^C (corrected for Suess effect) and δ^15^N ranges, and the correlation coefficients between these two stable isotope ratios

Salmon stock	Time range	Sampling frequency (# fish year^−1^) and Nbr. data point (*n*)	δ^13^C range (‰)	δ^15^N range (‰)	δ^13^C vs. δ^15^N
Kodiak Island	1967–1999	every year (2–5), last annulus only			
Red Lake^1^		*n* = 32	−19.05 to −17.17	8.29 to 11.62	0.22
Upper station		*n* = 31	−18.88 to −17.38	8.08 to 10.68	0.57***
SE Alaska	1968–1998	every year (2–5), last annulus only			
Chilkoot		*n* = 29	−18.92 to −17.43	9.46 to 11.66	0.67***
Chilkat		*n* = 29	−19.29 to −17.18	8.43 to 10.64	0.12
Columbia	1986–2016	every year (5)			
Okanagan		*n* = 31	−18.97 to −17.82	9.68 to 10.96	0.52**
Wenatchee		*n* = 31	−18.97 to −17.50	9.68 to 11.32	0.57***
BC coast	1960–2016	irregular (18–135)			
Rivers Inlet^2^		*n* = 16	−18.26 to −16.98	9.57 to 11.11	0.02
S Bristol Bay^3^	1962–2003	every 3rd year (3–5)			
Ugashik		*n* = 14	−18.13 to −16.98	10.47 to 12.03	0.37
Egegik		*n* = 15	−18.21 to −16.75	10.50 to 11.68	0.29
Naknek		*n* = 13	−18.04 to −16.79	10.48 to 12.22	0.69**
Kvichak		*n* = 11	−18.14 to −17.32	10.99 to 11.88	0.07
N Bristol Bay^3^	1962–2003	every 3rd year (3–5)			
Nushagak		*n* = 14	−17.87 to −16.80	10.89 to 12.76	0.06
Wood River		*n* = 14	−18.07 to −17.37	11.35 to 12.97	0.29
Igushik		*n* = 14	−18.44 to −16.62	10.79 to 12.14	0.16
Togiak		*n* = 15	−18.05 to −16.88	11.15 to 12.15	0.57*
NW Pacific	1995–2017	every year (3–5)			
Ozernaya		*n* = 23	−18.31 to −17.46	10.59 to 11.45	0.24
Kamchatka		*n* = 11	−18.33 to −17.39	10.45 to 11.48	−0.01

Note

Abbreviation:Nbr., number.

^1^Satterfield and Finney (2002); ^2^Espinasse et al. (2018); ^3^Johnson and Schindler (2012).

*p*‐values significance is: ***p < .001; **p < .01; *p < .05.

Kamchatka Peninsula salmon were collected at the river mouths, while salmon from Columbia River stocks were collected at Bonneville Dam about 230 km upstream from the sea. The Okanagan and Wenatchee time series were mixed until 2005 and identified separately afterward based on tag data. For four of the stocks processed (see Table 1), only the region between the last annulus and the periphery of the scale, which grows over the last year at sea, was processed. This part was excised from the rest of the scale following the description given by Satterfield and Finney (2002). Most of the scales were initially glued on gum cards. All the scales were immerged in water and rubbed thoroughly until the scales were transparent and free of residual glue. Details on stable isotope analysis of scales from SE Alaska and Kodiak Islands can be found in Satterfield and Finney (2002).

The scales processed during this study were dried in an oven for 24 hr at 60°C and sent for stable isotope analysis at UC Davis SIF (https://stableisotopefacility.ucdavis.edu/). The samples were analyzed for ^13^C and ^15^N isotopes using a PDZ Europa ANCA‐GSL elemental analyzer interfaced to a PDZ Europa 20–20 isotope ratio mass spectrometer (Sercon Ltd.). The system was calibrated using different NIST Standard Reference Materials. Measurement precision was assessed by running replicates of these standards and resulted in standard deviations consistently below 0.1‰ both for δ^13^C and δ^15^N. Isotopic ratios are expressed in the following standard notation:

$$\delta X\;=\;\left(\text{Rsample}\;/\;\text{Rstandard}\;‐\;1\right).$$

where X is ^13^C or ^15^N, and Rsample is the ^13^C/^12^C or ^15^N/^14^N, respectively. δ^13^C and δ^15^N were expressed in parts per thousand (‰) relative to external standards of Vienna Pee Dee Belemnite and atmospheric nitrogen, respectively.

The large amount of anthropogenic carbon dioxide released into the atmosphere has led to a long‐term decrease in both atmospheric and oceanic δ^13^C values, known as the Suess effect (Gruber et al., 1999). The extent of this decrease is directly linked to the rate of change of CO_2_ concentration and therefore has accelerated in recent decades (Swart et al., 2010). Analysis of δ^13^C time series should be corrected by adjusting values to a year of reference. We applied a correction factor of −0.02 ‰ yr^‐1^, in agreement with recent studies (Espinasse et al., 2018; Williams et al., 2007) and standardized the time series using 2015 as the year of reference.

C/N ratios are often used to correct δ^13^C values for the presence of lipids in the materials analyzed (Post et al., 2007). The scales of adult salmon are mainly made out of collagen and as such show constant C/*N* values varying between 2.5 and 2.9. However, for some of the published data (Bristol Bay and Rivers Inlet stocks), C/N was found out of this range. We suggest that the scales which are not rinsed directly after collection on fish might contain mucus residuals that will stick to the scale even when washed carefully before analysis. We applied a correction for the eight Bristol Bay stocks based on the difference between δ^13^C of scale with C/N > 3.5 and yearly average of δ^13^C scales having a C/N < 3.5. This resulted in correcting values for 65 scales out of 543 with a maximum correction factor of 1.2 ‰. The correction factor used for Rivers Inlet stock is also based on the differences in δ^13^C values between scales with expected C/N and scales with relatively high C/N (Espinasse et al., 2018) (Figure S1.1). It has been questioned if the scales should be acidified prior stable isotope analysis as the external layer of the scale is comprised of mineral apatite that could potentially skew analyses of δ^13^C (Tzadik et al., 2017). However, when the scales grow through the fish life cycle, new layers of collagen are added and the contribution of the external mineral layer to the total weight of the scale decreases (Hutchinson & Trueman, 2006). Furthermore, Sinnatamby et al. (2007) found no significant differences between δ^13^C values of Atlantic salmon scales that were acidified or not. Therefore, none of the scales processed during this study were acidified.

SST data were extracted from the COBE SST2 dataset, which can be downloaded freely at https://www.esrl.noaa.gov/psd/. This dataset provided SST interpolated on a 1 by 1 degree grid, and used a new analysis scheme to reduce uncertainties in analyzed SST (Hirahara et al., 2013). The mean of SST data was calculated for each grid cell from January to June, which generally provides highest correlation coefficients for both whole and excised scales. This corresponds to the time period during which sockeye salmon build up their reserves before migrating back to the spawning grounds.

### 2.2 Time series processing

For each stock, Pearson correlation coefficients were calculated at each grid point correlating associated SST time series and salmon δ^13^C time series, the latter consisting of the mean of δ^13^C values for each year. Correlation of time series can be caused by similarity in variations occurring at different frequencies. To be able to identify the relative effect due to long‐term trends (low frequency) and interannual variability (high frequency), three types of data were used: original data (combination of both effects), detrended data (only interannual variability), and smoothed data (dominance of long‐term trend). Time series subject to low‐frequency influence are usually strongly auto‐correlated, which violates the assumption of serial independence required for correlation test. One way to account for autocorrelation is to re‐assess the number of degrees of freedom, which results in higher *p*‐value for the same correlation coefficient. Pyper and Peterman (1998) developed the so‐called “modified Chelton method” that allows one to calculate a critical correlation coefficient value associated with a given *p*‐value. This method was later adapted by Barker et al. (2016) to account for missing values in time series. We applied this approach for calculation of the *p*‐values for the correlation test of original and smoothed data. In summary, the different types of processes applied to the data can be summarized as follows. For the smoothed data, both time series, δ^13^C and SST, were smoothed when the stocks were resolved yearly. For the other stocks, only the SST was smoothed. Smoothing of the data was conducted using the LOESS method with a span of three datapoints and one‐degree polynomial. Concerning the de‐trending process, the data were fitted with a linear regression and the residuals were used as detrended data. This was conducted on both time series, δ^13^C (after Suess correction) and SST, regardless the stock. Original data were only corrected for Suess effect. The map plotting was done with the Matlab package M_Map (Pawlowicz, 2019).

### 2.3 Area restriction and result validation

Initial plotting of the correlation coefficient between δ^13^C and SST can display potential spurious correlations due to inappropriate time series (small dataset or irregular sampling) or synchrony in SST variation across the North Pacific. A range of methods were explored to narrow down the area with highest correlation and propose the most realistic salmon distributions. The main source of information on salmon distributions in the literature comes from the tagging studies conducted by the three treaty nations (Japan, USA, Canada) of the INPFC from 1956 to 1970 (French et al., 1976). More than 60,000 sockeye salmon x.2 (two years spent at sea) or older were caught and tagged over this time period. Subsequently, the North Pacific Anadromous Fish Commission (NPAFC) has pursued the effort and has made tagging data available on request. The tag recovery data were used to produce distribution maps of maturing sockeye, a caveat being that since the fish were caught and tagged between late April to June, they were potentially already heading to their natal river system. It should also be noted that tagging data for a specific river system were only available for a limited number of years, and sometimes were not available. Stock‐specific data were used whenever available to assess the validity of the isotope‐based feeding ground estimates. At a broader scale, we digitized the regional distribution information presented in French et al. (1976) and included them as supplementary materials (Figure S1.2). Another approach to indirectly validate the proposed feeding grounds is to compare the average level of δ^13^C and δ^15^N values for stocks supposedly distributed in the same area and/or to test for δ^15^N correlation between stocks (assuming that stocks with the same at‐sea distribution will have correlated δ^15^N series). A prerequirement for the latter is to check that δ^13^C and δ^15^N values are not correlated. Pearson correlation coefficients between δ^13^C and δ^15^N time series were therefore calculated for each stock.. Finally, we used an empirical orthogonal function (EOF) analysis to describe the spatial mode of SST variability. EOF is a principal component analysis (PCA) but applied to spatial data. It allows identification of areas where the variable considered, that is, SST in this case, varies in a similar way over the period covered by the time series. This is useful to explain why we observed several discrete areas with high δ^13^C/SST correlation values.

The mean δ^15^N values of all salmon stocks found to be distributed in the NE Pacific were compared with δ^15^N baseline values published in Espinasse et al. (2019). In that study, the authors produced δ^15^N isoscapes (spatial distribution of δ^15^N values) based on zooplankton samples. The δ^15^N values were extracted from the isoscapes at a location centered on the highest likelihood distribution of each stock and averaged over one‐degree latitude and longitude. Stock‐specific trophic level was estimated assuming a trophic enrichment factor of 3.4‰ (Post, 2002).

## 3 RESULTS

### 3.1 Stock‐specific stable isotope values

**Figure 2:**
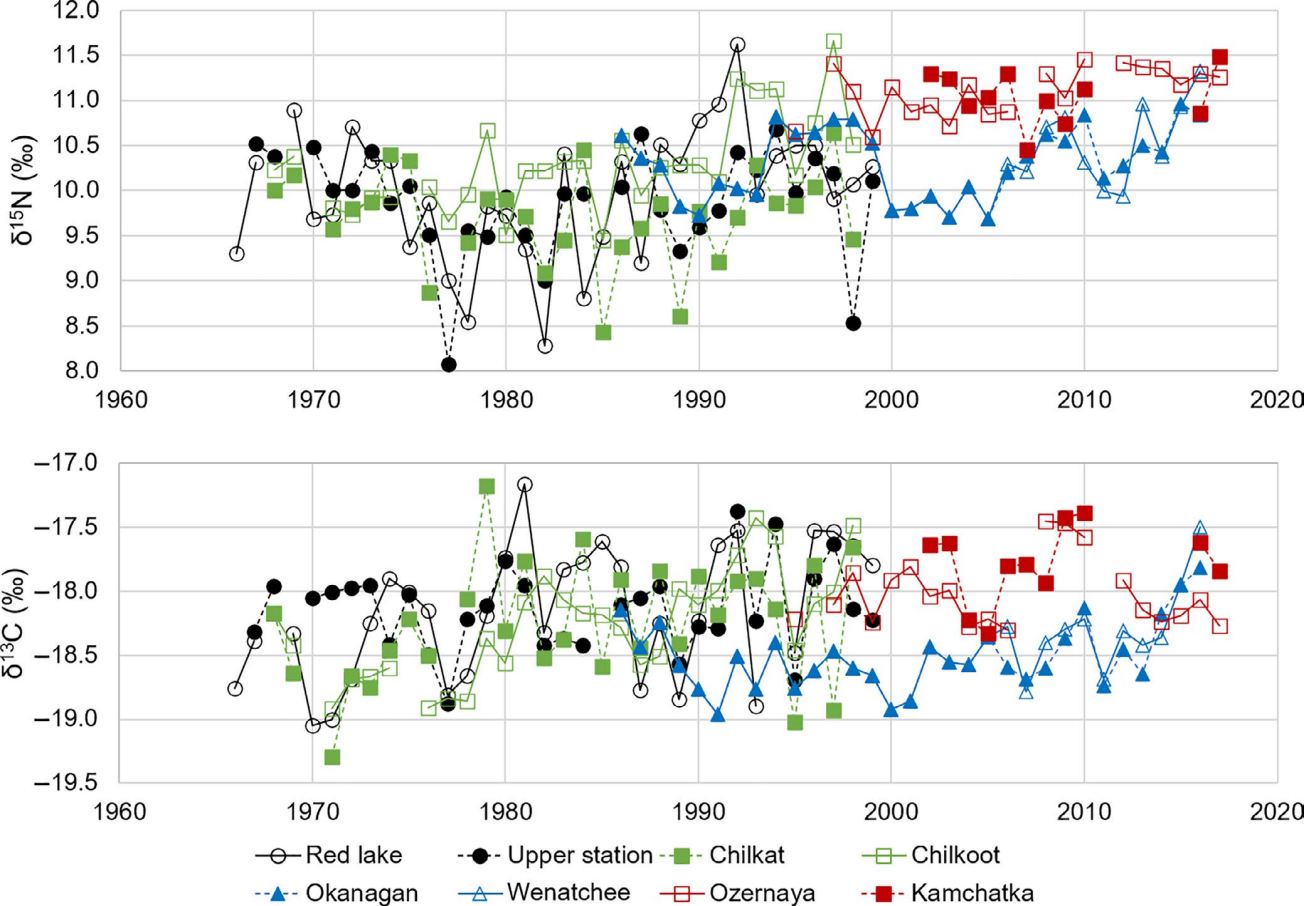
Yearly mean of δ^15^N and δ^13^C values (corrected for Suess effect) in scales of eight sockeye salmon stocks from Kodiak Island (Red Lake and Upper station), SE Alaska (Chilkat and Chilkoot), Columbia River (Okanagan and Wenatchee) and Kamchatka Peninsula (Ozernaya and Kamchatka)

Large interannual variations were observed for both δ^13^C and δ^15^N (Figure 2). Stocks that are in close geographic proximity showed similar patterns of low‐frequency stable isotope value variability but their stable isotope values differed substantially over small temporal scales. Suess effect‐corrected δ^13^C values showed more stability over time than δ^15^N and also less variation between stocks with values mainly distributed between −19 and −17.5‰ while δ^15^N values spanned over 3‰ (8.5‰ to 11.5‰).

**Figure 3:**
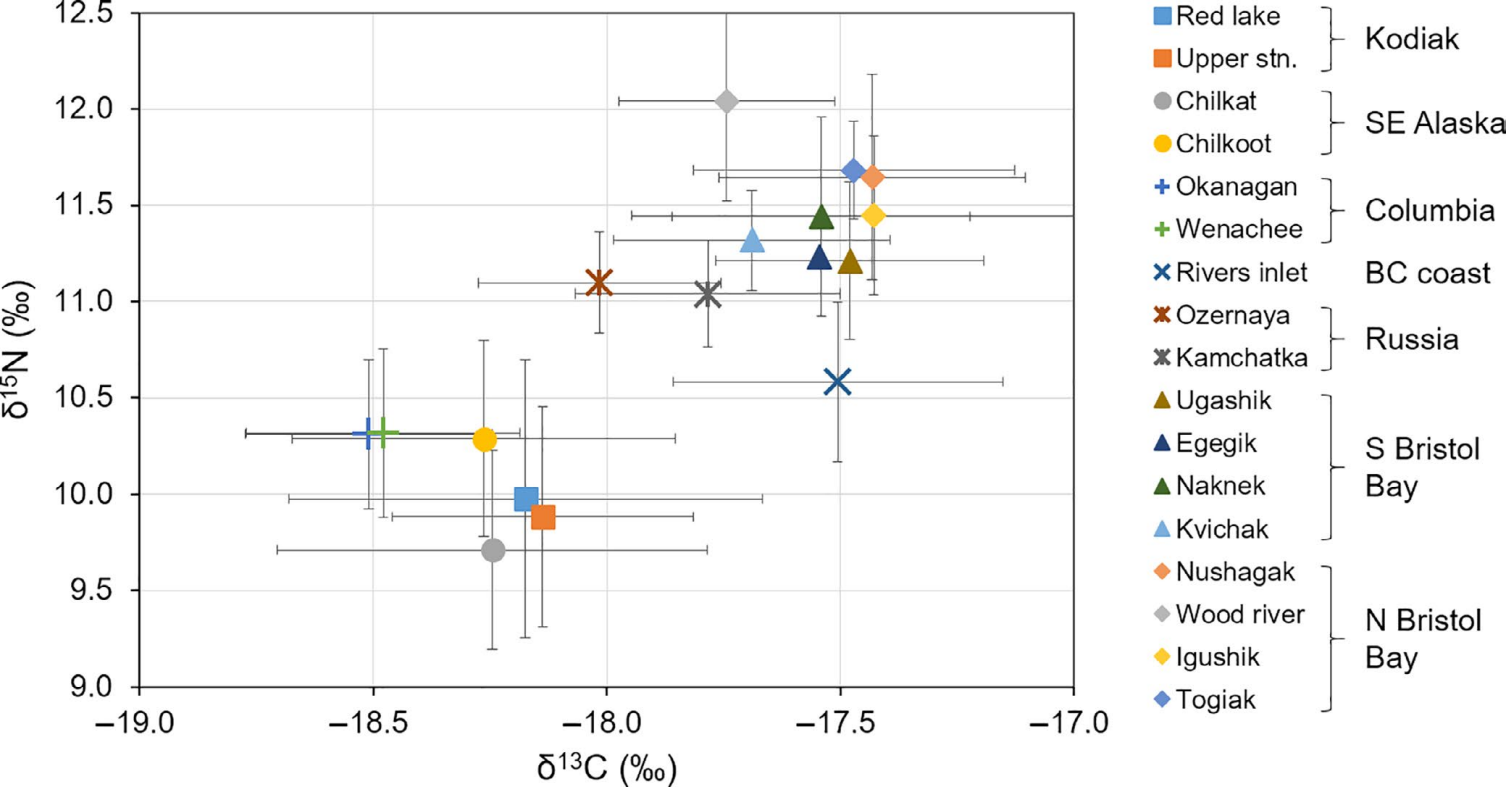
Biplot of δ^15^N vs. δ^13^C values (corrected for Suess effect) averaged over different time periods (see Table 1) for 17 sockeye salmon stocks. The error bars represent the standard deviation

For clarity, only unpublished data (with the addition of the Red Lake stock) were represented in Figure 2, and however, the assessment stands for all stocks (Table 1) with the absolute range being wider for δ^15^N than δ^13^C values, 5.05 vs. 2.67, respectively. Significant correlation between δ^15^N and δ^13^C appeared random among stocks with no obvious pattern in region or age. The average of stable isotope values for each stock showed clear separation between the main regions despite the differences in time range covered (Figure 3). Kodiak Island and SE Alaska stocks together with Columbia River stocks showed lower δ^15^N and δ^13^C values. All Bristol Bay stocks grouped together with higher δ^15^N values, while Russian stocks and BC coast stocks were positioned between these aforementioned groups.

### 3.2 Stock‐specific feeding grounds

**Figure 4:**
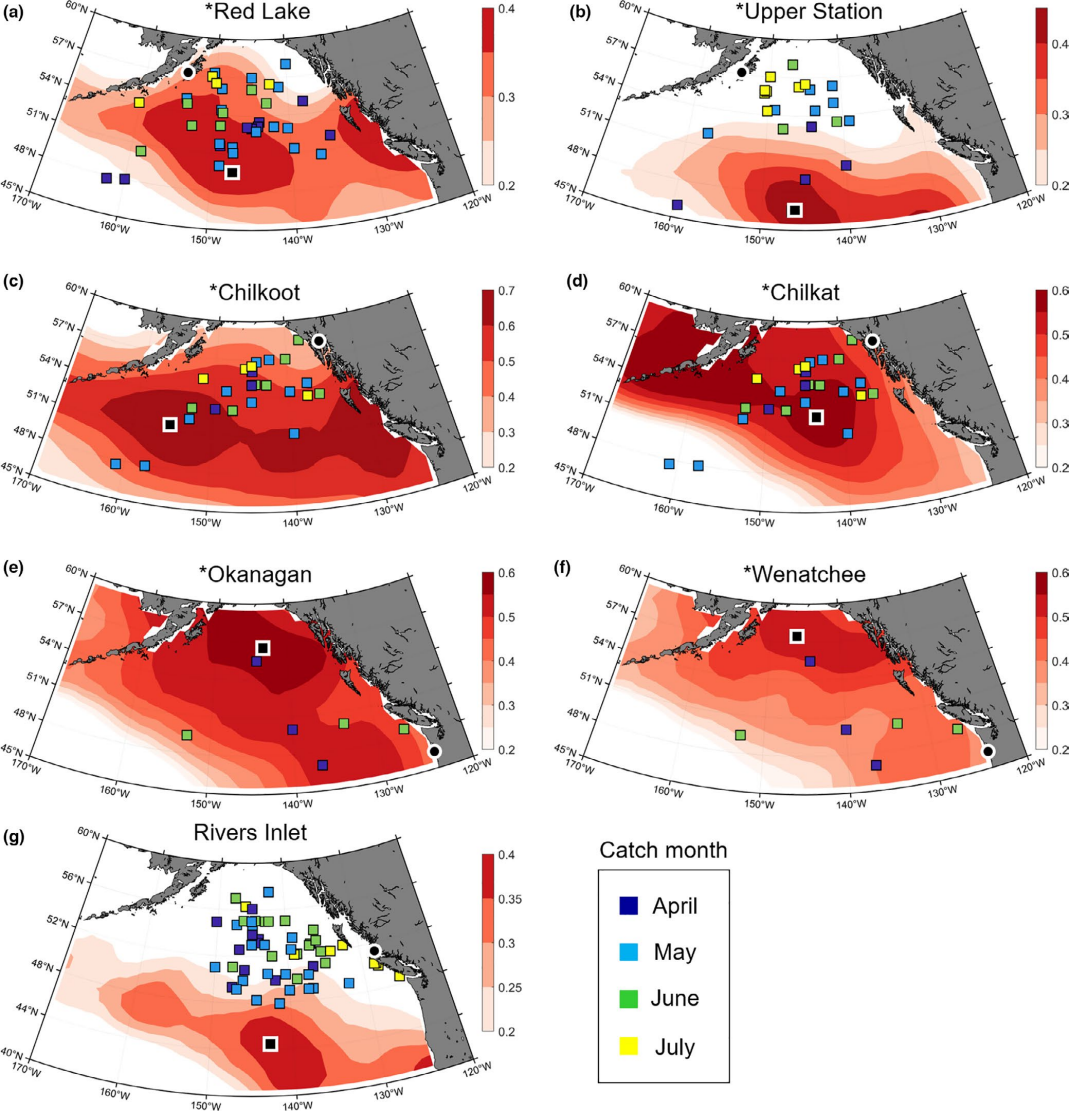
Estimated feeding grounds during the last 6 months at sea of (a) Red lake, (b) Upper station, (c) Chilkoot, (d) Chilkat, (e) Okanagan, (f) Wenatchee, and (g) Rivers Inlet sockeye salmon stocks (Gulf of Alaska). The black squares with white border indicate the most likely feeding ground based on tagging data and the strength of the correlation between SST and δ^13^C. Significant correlations are indicated by an asterisk in front of the stock name. Colored squares represent tagging data with color varying from April to July depending on the release month. Locations of the point of entry in the freshwater system of the different stocks are shown (black disk bordered in white)

Maps of the correlation between SST and δ^13^C time series were interpreted with respect to tagging data (*n* = 266; 95% of them recorded before 1970). In the database, Chilkat/Chilkoot data were pooled together and Red Lake/Upper Station tagging recovery areas encompass not only the river system but also larger surrounding coastal area. The proposed feeding grounds for stocks with spawning grounds south of the Aleutian Islands were all located in the NE Pacific itself but with little overlap between the stocks (Figure 4). The SE Alaska stocks Chilkat and Chilkoot, which have spawning grounds 10 km away from each other, suggested well‐separated at‐sea locations, with Chilkoot being distributed 10 degrees farther west than Chilkat. While Columbia River stocks have the most southerly spawning grounds, their high‐seas distributions were located in the northern sector of the NE Pacific. The Rivers Inlet stock was distributed farther south than any other stock, differing from the northerly distribution apparent in the tagging data.

**Figure 5:**
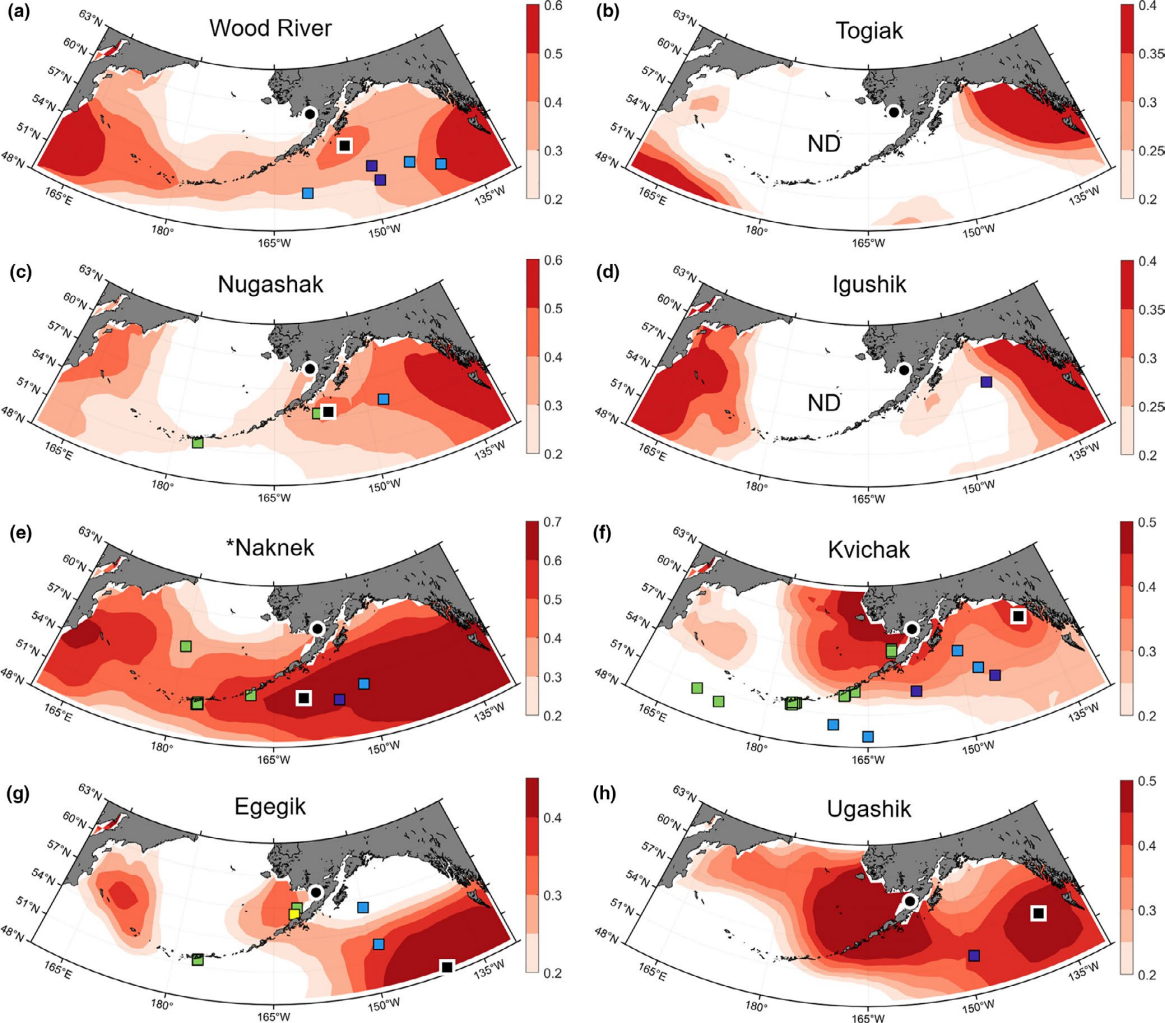
Estimated feeding grounds during the last 6 months at sea of (a) Wood River, (b) Togiak, (c) Nugashak, (d) Igushik, (e) Naknek, (f) Kvichak, (g) Egegik, and (h) Ugashik sockeye salmon stocks (Bristol Bay from north to south). The black squares with white border indicate the most likely feeding grounds based on tagging data and the strength of the correlation between SST and δ^13^C. Significant correlations are indicated by an asterisk in front of the stock name. Colored squares represent tagging data with color varying from April to May depending on the release date (see legend in Figure 4). Locations of the point of entry in the freshwater system of the different stocks are shown (black disk bordered in white). ND, not determined

With the exception of Rivers Inlet, all stocks were sampled yearly and produced significant correlations with highest significance usually found using the original time series (Table 2, correlation coefficients were usually higher with smoothed time series but the adjusted degrees of freedom reduced the significance, see Methods). Bristol Bay stocks at‐sea distributions showed interesting patterns with some stocks being distributed along the Aleutian Islands and others farther to the east in the Gulf of Alaska (Figure 5).

**Table 2:** Correlation coefficients between δ^13^C values in salmon scales of several stocks and SST (averaged for January–June of the year of return)

Salmon stock	Correlation coefficient		
	Original	Detrended	Smoothed
Kodiak Island			
Red Lake	0.42*	0.25	0.36
Upper station	0.44**	0.42*	0.60**
SE Alaska			
Chilkoot	0.72**	0.47*	0.86*
Chilkat	0.58***	0.57***	0.69*
Columbia			
Okanagan	0.59**	0.55**	0.73**
Wenatchee	0.54**	0.47**	0.69**
BC coast			
Rivers Inlet	0.38	0.62*	–
S Bristol Bay			
Ugashik	0.49.	0.46.	0.36.
Egegik	0.48.	0.37	0.35
Naknek	0.67*	0.51	0.66*
Kvichak	0.42	0.27	0.30
N Bristol Bay			
Nushagak	0.42	0.27	0.35
Wood River	0.46.	–	0.52.
Igushik	–	–	–
Togiak	–	–	–
NW Pacific			
Ozernaya	0.43	0.44	0.63*
Kamchatka	0.41	0.43	0.60

**Figure 6:**
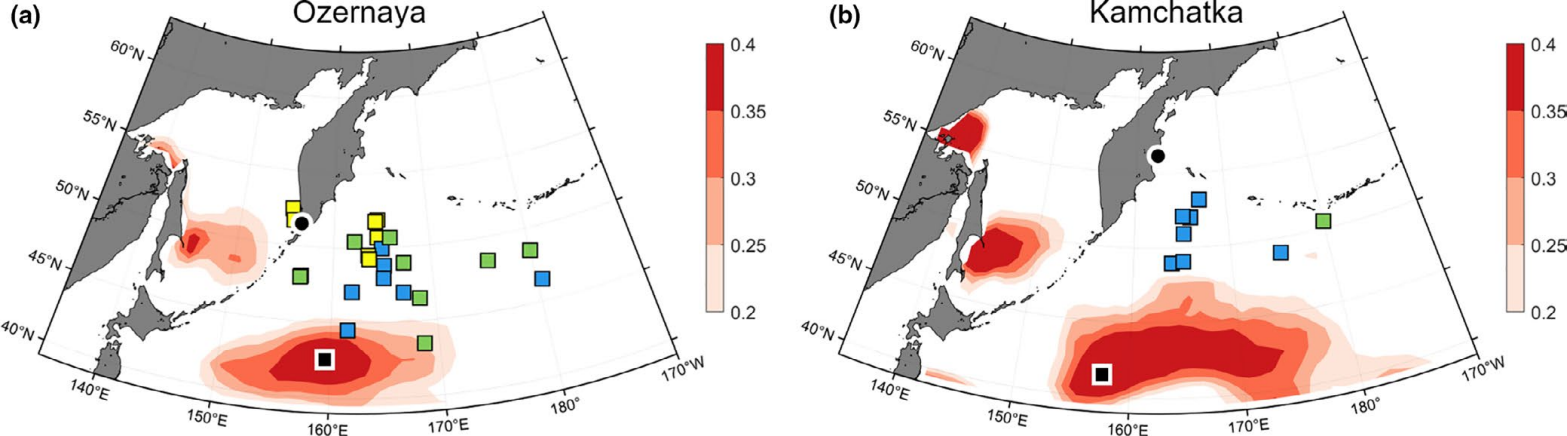
Estimated feeding grounds during the last 6 months at sea of (a) Ozernaya and (b) Kamchatka sockeye salmon stocks (Russia). The black squares with white border indicate the most likely feeding grounds based on tagging data and the strength of the correlation between SST and δ^13^C. Significant correlations are indicated by an asterisk in front of the stock name. Colored squares represent tagging data with color varying from April to May depending on the release date (see legend in Figure 4). Locations of the point of entry in the freshwater system of the different stocks are shown (black disk bordered in white)

The time series were processed in different ways to disentangle the effect of low‐ and high‐frequency variability on correlation estimates (original, detrended, and smoothed). SST data for each stock were retrieved at the location marked in Figures 4, 5 and 6 by a black square. *p*‐values were calculated using the modified Chelton method to account for autocorrelation.

*p*‐values significance is: ***p < .001; **p < .01; *p < .05 and.*p* < .1.

However, low correlation coefficients, mostly nonsignificant, prevented us from proposing feeding grounds for all stocks. This was most likely the result of the every 3‐year sampling resolution that resulted in a low number of data points that did not capture the full extent of interannual variability. Both Russian stocks showed similar and uniquely more western at‐sea distributions despite originating from opposite sides of the Kamchatka Peninsula (Figure 6). Smoothed time series provided more significant correlations for these stocks.

**Figure 7:**
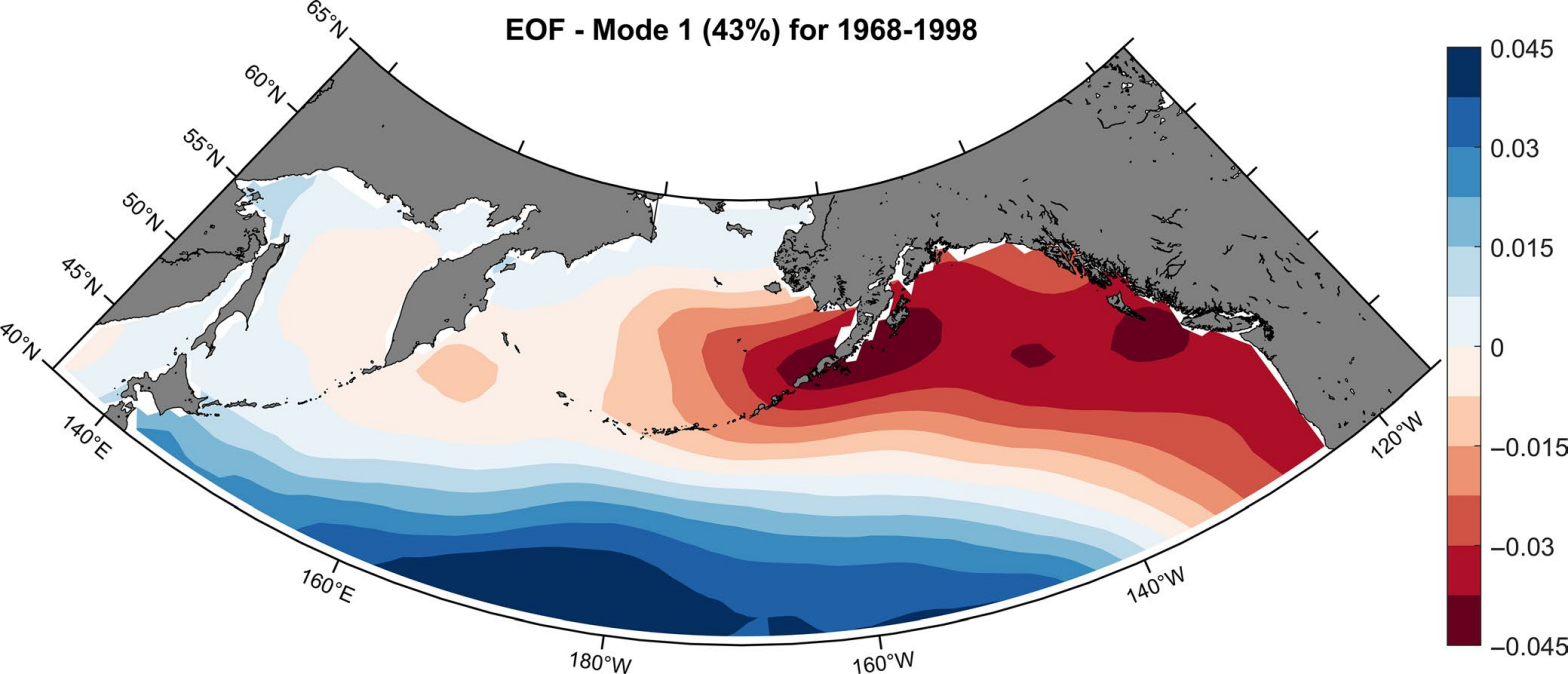
First mode of an empirical orthogonal function (EOF) analysis using SST averaged each year from January to June for 1968–1998. The EOF identifies areas where SST had a similar pattern of variability over the period of the time series. The first mode of the EOF explained 43% of the variance

The first mode of the EOF analysis applied to SST data for 1968–1998 explained 43% of the total variance and showed that waters from SW Alaska and North BC coast covaried (Figure 7).

**Figure 8:**
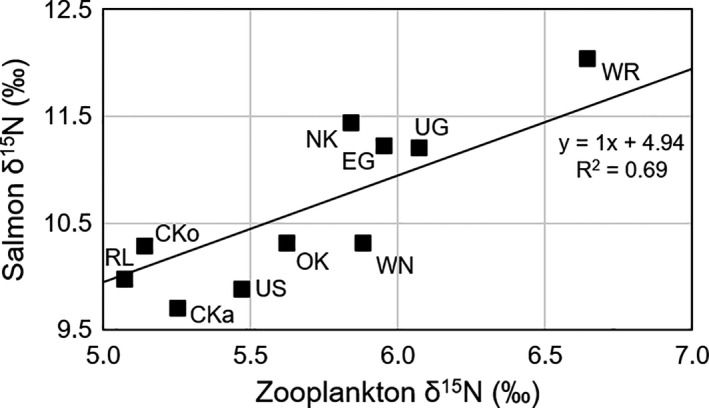
Salmon δ^15^N plotted against zooplankton δ^15^N values. Each point represents an average value for a sockeye salmon stock that have been assessed to be distributed in the Gulf of Alaska. Zooplankton δ^15^N estimates were obtained from isoscapes presented in Espinasse et al. (2019). CKa, Chilkat; CKo, Chilkoot; EG, Egegik; NK, Naknek; OK, Okanagan; RD, Red Lake; RI, Rivers Inlet; Ug, Ugashik; US, Upper Station; WN, Wenatchee; WR, Wood River

The feeding grounds of eleven of the salmon stocks were estimated to be located in the NE Pacific. We compared the averaged δ^15^N values of these stocks with δ^15^N values extracted from zooplankton isoscapes (Espinasse et al., 2019) (Figure 8). The linear regression applied to these data gave a Y intercept value of 4.94 (with zooplankton as TL = 2), with the coefficient set to 1. Assuming a trophic enrichment factor of 3.4‰, the trophic levels of the salmon stocks varied between 3.30 and 3.59 (Table 3).

**Table 3:** Trophic level estimates for 11 salmon stocks assessed in this study to be distributed in the Gulf of Alaska during their last 6 months (January–June) at sea. The trophic level is based on the difference between δ^15^N values in salmon scales and large herbivorous copepods (TL 2) analyzed for this region, assuming a trophic level enrichment factor of 3.4‰

Salmon stock	Trophic level
Columbia	
Okanagan	3.38
Wenatchee	3.31
SE Alaska	
Chilkoot	3.51
Chilkat	3.31
Kodiak Island	
Red lake	3.44
Upper station	3.30
BC coast	
Rivers Inlet	3.34
Bristol Bay	
Naknek	3.55
Wood River	3.59
Ugashik	3.51
Egegik	3.55

## 4 DISCUSSION

The goal of this study was to estimate the high‐seas distribution and foraging grounds of sockeye salmon. We analyzed 17 sockeye salmon stocks and found significant correlations between SST and δ^13^C for 7 of them. The spatial pattern of correlation between SST and δ^13^C did not always result in the identification of a single well‐defined foraging region. The accuracy of the results relies on the assumptions that (a) the fish forage in a similar location every year and (b) that they do not move across a strong SST/prey δ^13^C gradient during the time period over which the isotopic composition is integrated.

The first assumption is difficult to fully assess given that there are limited data available on high‐seas stock‐specific locations. Among the stocks considered in this study, Rivers Inlet is the stock for which the amount of tagging data is the largest (*n* = 60 for Apr–Jun). From 1962 to 1967, the yearly latitudinal and longitudinal means span from 51.45 to 53.05°N and from 138.67 to 144.63°W, respectively. Based on this single stock, the fish seemed to show a tendency to return to a similar area every year. Although this provides evidence for a consistent high‐seas distribution, these data for a single stock cannot be reliably applied to other stocks. However, it has been shown that salmon may reach their feeding grounds using magnetic fields and that the “magnetic map” leading to their feeding grounds may be inherited (Putman et al., 2014). This suggests that stock‐specific feeding grounds will have consistent geographic locations irrespective of ocean conditions.

This question is probably even more essential for stocks with a strong pattern in age distribution (one predominant age‐class), resulting in little mixing between cohorts from different brood years. Another aspect that should be investigated is whether there are differences in δ^13^C values between early and late runs for the same stock. While for some stocks, adult salmon return predominantly occurs over a short time window, other stocks have two temporally separated runs identified and it is not currently known if high‐seas distributions differ between runs.

The second assumption is also difficult to evaluate as no data are available for stock‐specific movement. A strong gradient in δ^13^C values at lower trophic levels is usually located at the transition between the coastal system (higher productivity, subject to fluvial input, resuspension, etc.) and the open ocean system (more oligotrophic conditions, Fe‐limited). This transition usually occurs above the shelf or continental slope, depending on the width of the shelf, local hydrodynamics, and water depth, but in all cases occurs at a relatively short distance from the coast (El‐Sabaawi et al., 2012; Kline, 2009). Maturing salmon will likely only swim through this zone once, just before entering their river system to spawn, and it is therefore unlikely to influence their δ^13^C values. They could, however, target areas of higher productivity while in the high seas. For example, eddies have the potential to enhance local primary production that will promote development of salmon prey (Crawford et al., 2007; Mackas et al., 2005) with higher δ^13^C values (Espinasse et al., 2019). Unfortunately, it is not yet documented whether the salmon display such behavior.

Aside from these two assumptions, other factors not directly related to SST can affect carbon isotopic composition and represent a source of variation in δ^13^C values between years that we were unable to account for in this study. Isotope fractionation at the base of the food web can be influenced by several factors related to phytoplankton community composition (Burkhardt et al., 1999; Hoins et al., 2016), growth rate (Burkhardt et al., 1999; Riebesell et al., 2000), cell geometry (Burkhardt et al., 1999; Popp et al., 1998), or carbon sources (Hoins et al., 2016). The fractionation factor has also been shown to change over time under anthropogenic influence (Young et al., 2013). Finally, the trophic structure and interactions among lower trophic levels will determine how the isotopic composition translate from autotrophs to salmon prey (Caut et al., 2009). These factors, together with uncertainties associated with the two previously described assumptions and the limits of some of the time series (e.g., resolution, length), may all have contributed to not finding significant correlations for all the stocks.

### 4.1 Influence of scale sampling method

Our dataset comprised a mix of δ^13^C and δ^15^N values measured from whole scales and the last annulus of the scale. We integrated SST over different time ranges to evaluate the best match with the potential integration time of δ^13^C values from these different methodologies. The time period from January to June of the return year gave the best results for the two scale sampling methods. This suggested a negligible influence of scale material laid down during the freshwater phase (Hutchinson & Trueman, 2006) and furthermore that the whole scale δ^13^C value was dominated by the last growing season. This can be explained by the largest increase in size and weight of the fish occurring during the last year at sea (Ishida et al., 1998). The mass of collagen produced during this time period largely overwhelms the rest of the scale mass. Our results suggest that using the whole scale did not affect time series comparison, as whole scale and last annulus have very similar stable isotope values (B. P. Finney, unpublished data). Studies focusing on the freshwater phase or early marine phase of salmon should, however, be careful in their interpretations when using scales from maturing fish, as it seems inevitable that the recent layers deposited will strongly influence the stable isotope values (Hutchinson & Trueman, 2006).

We demonstrated that when analyzing time series of >20 data points with yearly resolution, using the original data (only corrected for Suess effect) gave the most significant results. These data retain the influence of both low‐ and high‐frequency variations but require correction of the number of degrees of freedom to adjust the *p*‐value (Pyper & Peterman, 1998). Some stocks with geographically close spawning grounds showed δ^13^C variations driven by different factors. For example, Chilkoot salmon δ^13^C data demonstrated a largely increasing trend from low values in earlier years to high values in later years, while Chilkat did not show a clear long‐term trend but rather greater interannual variability (Figure S1.3). The Suess effect correction parameter, by changing the overall inclination of the δ^13^C trend, could affect the part of the correlation driven by the low frequency (long‐term trend). The uncertainties associated with how the Suess effect varies with latitude, between regions and over time, makes it difficult to set a universal factor (Eide et al., 2017; Young et al., 2013). We tested the sensitivity of our approach to the Suess effect correction parameter using data for the Chilkoot and Chilkat stocks, and additionally the Okanagan stock for which the data covers more recent years. In these tests, the Suess effect parameter was set at −0.015 and −0.025‰ year^‐1^. Both parameter values resulted in similar distributions and very little change in correlation significance (Table S1.1). Furthermore, for the large majority of the stocks for which we found significant correlations, the three types of series generated the same distribution. Among the series with yearly resolution, only Red Lake displayed different high correlation areas between the original series and the smoothed series. The detrended series, however, gave a similar, but nonsignificant, distribution to the original one. Overall, using the three approaches enabled improved evaluation of the ocean area most likely used by the salmon, helping avoid locations which were only driven by interannual variability or long‐term trend.

Series with coarser or irregular resolution, usually the smaller datasets (<20), gave mixed results in defining feeding grounds. While δ^13^C time series for Russian stocks were sufficient to suggest realistic spring feeding grounds in the western Pacific despite limited number of datapoints, results for Bristol Bay showed more contrast between stocks, with some stocks showing uncertain distributions. This was potentially due to the 3 years resolutions used for these times series. Smoothing the SST series helped to increase the significance of the correlation tests but also created high correlation areas that were later dismissed based on tagging data. We tested the effect of sampling resolution by downgrading the resolution of the Okanagan series using data from every 3rd year and obtained comparable distributions to the full resolution dataset (Figure S1.4). However, we do not encourage the use of small datasets or low resolution/irregular time series, as it is often hard to estimate if the series meets the basic assumptions for a correlation test, and there is an increased chance of spurious correlations.

### 4.2 Feeding grounds definition and validation

While for some of the stocks, such as Chilkoot, Okanagan, and Wenatchee, the method resulted in well‐defined feeding ground estimates, estimates for other stocks showed more uncertainty with larger areas showing high correlation and/or multiple regions with high correlation. Several approaches were used to assess these areas and to further resolve the feeding grounds. Regional data from tagging studies were used to define the likely large scale distributions of salmon (Figure S1.2). However, these distributions encompassed large areas as many stocks were pooled, and so these data were mainly useful to discard high correlation spots that were located outside the potential distribution range. For example, for Red Lake and Upper Station stocks (Kodiak Island), two secondary high correlation spots were located eastward of the range determined by the tagging studies and were therefore not considered reasonable. Stock‐specific tagging data were used to recreate salmon migration pathways to their native river system, assuming that their locations in early months, that is, April and May, corresponded with their spring feeding grounds. One caveat associated with the comparison between the tagging‐derived and isotope‐derived feeding grounds is that the vast majority of the tagging data were collected before 1970 while most δ^13^C time series cover more recent times (Table 1). Also, in most cases tagging data were not numerous enough to allow a clear pattern to emerge.

The EOF analysis of SST data was useful to understand how water mass temperature varied over time, independently of the correlation with δ^13^C values. The Chilkat stock distribution was a good example, with a high correlation area covering a spot in the middle of the Gulf of Alaska and extending northwest to Alaska. This may have been due to the SST of these water masses varying in a similar way during the time period considered for this stock (Figure 7). Based on the tagging data, the central Gulf of Alaska region was estimated to be more realistic. Similarly, most Bristol Bay stock distributions showed relatively high δ^13^C/SST correlations values along the Canadian coast. We suggest that the wider range of temperature occurring in coastal areas, combined with limited datapoints increased the chances of obtaining spurious correlation and, based on the few tagging data available, this area can be dismissed as a potential feeding ground.

Another way to validate the inferred stock distributions is to integrate information provided by the δ^15^N values. We would expect that the stocks with similar foraging areas would also show correlation between their δ^15^N time series, assuming that they feed at the same trophic level. However, very few stocks showed significant correlation of δ^15^N values (Figure S1.5). One explanation for this is that there was little overlap between stock distributions. Differences in return timing may also have been a factor, since the δ^15^N values of prey change rapidly over the productive season (Kline, 1999). Based on average levels of δ^15^N and δ^13^C (Figure 3), Bristol Bay stocks were separated from the others by their higher stable isotope ratios. This was in agreement with the distributions of the stocks for which the spring feeding grounds were clearly defined and matched relatively well with tagging data (Ugashik, Naknek and Wood River). These stocks were distributed closer to the coast of the Aleutian Islands or the SE Alaskan coast (Figure 5), and both of these areas are more productive and display elevated stable isotope baselines (Espinasse et al., 2019; Pomerleau et al., 2014). Considering the NE Pacific, Rivers Inlet stock distribution stood out with higher δ^13^C values and to a lesser extent δ^15^N values than other stocks, which was well explained by the proposed feeding grounds located quite far south (Figure 4). This is also further supported by the atypical migration behavior of Rivers Inlet juvenile salmon that, in contrast to others stocks, do not seem to migrate northward in their first summer at sea (Beacham et al., 2014). However, the tagging data did not validate this location. Since all tagging data related to this stock were recorded between 1962 and 1967, it is a possibility that the stock distribution shifted after its dramatic collapse in the early 1970s (McKinnell et al., 2001) and the important climate shift in 1977 (Hare & Mantua, 2000).

Comparison of δ^15^N mean values for stocks with geographically close spawning grounds also seem to support the proposed feeding grounds. While Okanagan/Wenatchee stocks and Red Lake/Upper Station stocks were estimated to be distributed relatively close to one another and indeed showed similar δ^15^N values, Chilkat/Chilkoot stocks differed both in their spatial distribution and δ^15^N values (Figure 3).

We found a positive correlation between the δ^15^N of salmon prey and salmon for the stocks with feeding grounds identified in the Gulf of Alaska. This correlation was mainly driven by differences in δ^15^N values observed between NE Pacific stocks and Bristol Bay stocks. However, the fact that, despite the poor time series resolution for the Bristol Bay stocks, the proposed feeding grounds were located in areas that were assessed to have a higher δ^15^N baseline than Gulf of Alaska stocks, lends support for our results. This also allowed estimation of the trophic level for these stocks (Table 3). Salmon are reported to feed on a large variety of prey such as copepods, squids, euphausiids, and amphipods (Kaeriyama et al., 2004), and therefore, one would except their trophic level (TL) to range between 3 and 4, which is consistent with our estimates (TL = 3.3–3.8) and those from Qin and Kaeriyama (2016) (TL = 3.9).

### 4.3 Implications

A broad regional approach, assuming a generalized distribution for all salmon stocks in the North Pacific, has been demonstrated to be useful in linking salmon productivity with climate indices (Malick et al., 2017; Mantua et al., 1997). However, other studies have stressed the importance of stock‐specific analysis, as stocks can have different productivity trends despite having geographically close spawning grounds (Quinn et al., 2012; Rogers & Schindler, 2011), and there is a need to differentiate between factors affecting salmon at local to regional scales (Ohlberger et al., 2016). The unique stable isotope signatures for each stock considered in this study imply different feeding locations. A similar finding has previously been reported in other regions (MacKenzie et al., 2012, 2012; Torniainen et al., 2014). Knowledge of stock‐specific at sea distributions is likely to contribute to better understanding stock‐specific productivity trends. For example, there are still uncertainties associated with at sea mortality rates and how it affects year to year production. In the North Atlantic, based on the same approach, feeding ground estimates for salmon were used to refine the causes of the ongoing population decline (Soto et al., 2018). As the fish that spent one or several winters in the high seas were distributed in different areas, characterized by contrasting trends in water temperature, warming cannot be the only explanation for the population decline. Changes in environmental conditions due to hydrographic or atmospheric processes impact the physical environment experienced by salmon and also the quality and/ or quantity of prey that they encounter. Because of the heterogeneity in their prey distribution, the high‐seas distributions of salmon can affect their productivity (Trueman et al., 2012).

At a large spatial scale, the difference in trends of productivity between Bristol Bay stocks and most of the NE Pacific stocks during the last decades may be explained by differences in high‐seas distributions. In this study, we found Bristol Bay stocks to be primarily distributed around the Aleutian Islands and farther south along the Canadian coast (Figure 5), while the NE Pacific stocks were distributed in the offshelf area of the Gulf of Alaska (Figure 4). These stocks would thus have experienced different environmental conditions with respect to both annual conditions and long‐term trends in factors such as SST (Mueter et al., 2002) and ocean currents (Malick et al., 2017).

At a regional scale, stock‐specific distributions can help explain divergence in productivity of stocks with geographically close spawning grounds. Peterman and Brigitte (2012) investigated similarity in temporal variation of productivity of several sockeye stocks and found that among Bristol Bay stocks, Wood River, Naknek, Togiak and Igushik stocks grouped together while Egegik and Ugashik formed another group, breaking the usual south/north geographical separation of the stocks. However, this grouping is consistent with the pattern of salmon at sea distributions found in this study, with Wood River, Naknek, and Togiak distributed along the south coast of the Aleutian Islands and Egegik and Ugashik farther south. This highlights our finding that stocks with similar spawning ground locations should not be assumed to have similar at‐sea distributions, and the need for at‐sea stock identification data to explicitly connect stocks to high‐seas conditions.

## 5 CONCLUSIONS

Given potential reductions in favorable habitat due to accelerating global warming, there is an urgent need to better understand the ocean environment experienced by salmon and how this impacts their health and condition. Up to now, little information has been available on stock‐specific salmon distribution which hampers our ability to resolve stock‐specific dynamics and develop appropriate management plans. The marine environment is heterogeneous, and populations foraging in different areas may be affected in different ways as environmental conditions change. The use of δ^13^C/SST relationship provided a complementary approach to tagging or genomic approaches, that unlike the latter methods does not require sampling fish at sea. Although the identification of spring feeding grounds by this method is based on many assumptions, it provides testable hypotheses to both verify stock feeding locations and mechanisms regulating population dynamics. The use of long‐term interannual time series gave reasonable estimates in many cases, which could be validated by δ^15^N data. Expanding this approach with data from other major sockeye stocks and other salmon species would improve understanding of the mechanisms explaining salmon spatial distribution in the high seas, and how this distribution interacts with environmental conditions to affect salmon survival and fitness.

## CONFLICT OF INTEREST

The authors declare that they have no competing interests.

## AUTHOR CONTRIBUTION

Boris Espinasse: Conceptualization (lead); Data curation (lead); Methodology (lead); Validation (lead); Writing‐original draft (lead); Writing‐review & editing (lead). Brian PV Hunt: Conceptualization (equal); Data curation (equal); Funding acquisition (lead); Supervision (equal); Validation (equal); Writing‐review & editing (equal). Bruce Finney: Conceptualization (equal); Data curation (equal); Validation (equal); Writing‐review & editing (equal). Jeffrey Fryer: Data curation (equal); Methodology (supporting); Validation (supporting); Writing‐review & editing (equal). Alexander V Bugaev: Data curation (equal); Methodology (supporting); Validation (supporting); Writing‐review & editing (equal). Evgeny A Pakhomov: Conceptualization (equal); Funding acquisition (equal); Supervision (equal); Writing‐review & editing (equal).

## AUTHORS' CONTRIBUTIONS

BE, BPVH, BPF, and EAP conceived the project. BPF, JKF, and AVB provided access to new materials (archived scale) or unpublished salmon stable isotope data. BE carried out the sample and data analysis and wrote the manuscript. All authors provided editorial advice. All authors read and approved the final manuscript.

## Supporting information

File S1

Supplementary Material

## Data Availability

All stable isotope data supporting the conclusions of this article have been deposited in the Dryad digital repository (https://doi.org/10.5061/dryad.hqbzkh1dw).
