# Supplementary material for: Using stable isotopes to infer stock‐specific high‐seas distribution of maturing sockeye salmon in the North Pacific
Source: Ecol Evol. 2020 Nov 13;10(23):13555–70. doi: 10.1002/ece3.7022 (PMC7713939; doi:10.1002/ece3.7022)
Supplement: Supplementary file 1 — File S1 [file ECE3-10-13555-s001.pdf]

Additional file 1

Table S1 Sensitivity test on correlation coefficients between $\delta^{13}C$ values in salmon scales of three sockeye salmon stocks and SST (averaged for Jan-Jun) by varying the Suess effect parameter. The time series were processed in different ways to disentangle the effect of low and high frequencies on correlation estimates (original, detrended and smoothed). SST data were retrieved at the location marked in Figures 4, 5 and 6 by a white cross. P-values were calculated using the modified Chelton method to account for autocorrelation. P-values significance is: *** p<0.001, **p<0.01, *p<0.05.

Salmon stock	Suess effect parameter	Correlation coefficient		
		original	detrended	smoothed
Chilkat	-0.015	0.53***	0.57***	0.62*
	-0.020	0.58***	0.57***	0.69*
	-0.025	0.63***	0.57***	0.75*
Chilkoot	-0.015	0.69**	0.47*	0.83*
	-0.020	0.72**	0.47*	0.86*
	-0.025	0.73**	0.47*	0.88*
Okanagan	-0.015	0.57**	0.55**	0.71**
	-0.020	0.59**	0.55**	0.73**
	-0.025	0.60**	0.55**	0.74**

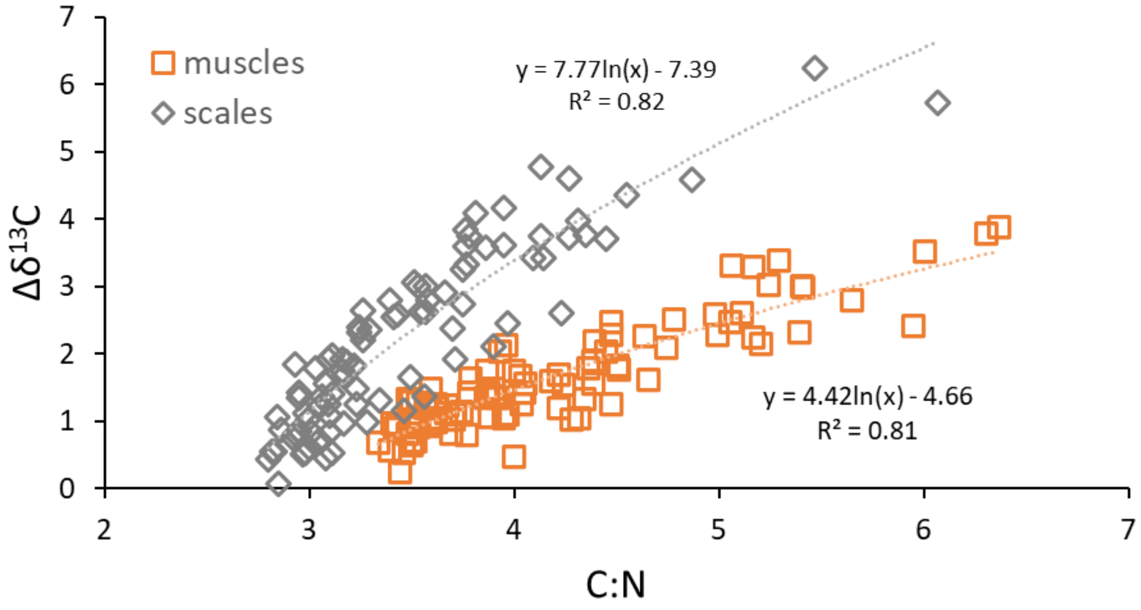

Figure S1. Relationship between CN ratios and $\delta^{13}C$ of 2013 pre-spawn and post-spawn Rivers Inlet sockeye scales and muscle tissue fitted using logarithmic models. $\delta^{13}C$ reference value for $\Delta\delta^{13}C$ calculation is the median of $\delta^{13}C$ distribution in muscles/scales of post-spawn salmon. It is unclear why the scales show a similar pattern than muscles, we suggest that in some situations (poor immediate washing) surrounding mucus could soak into the scales.

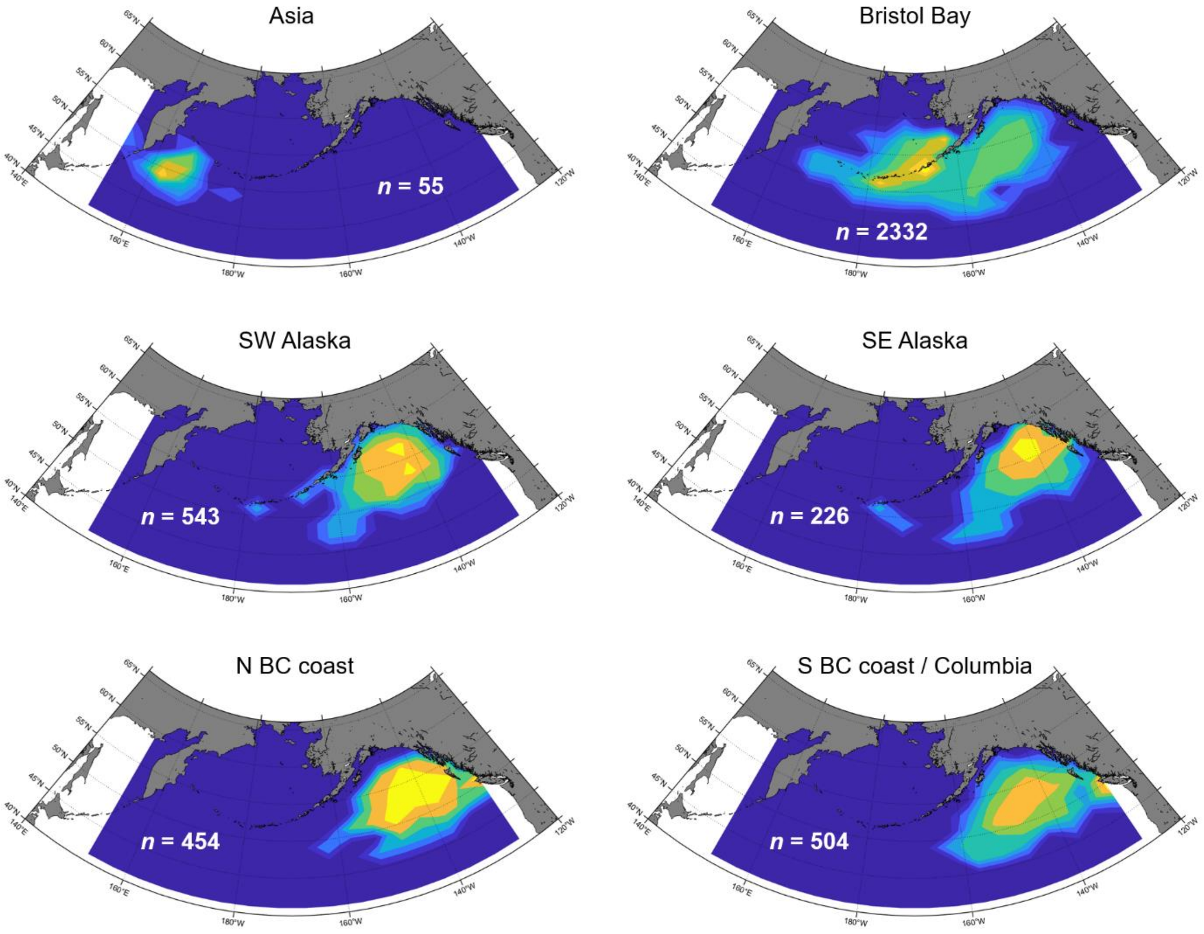

Figure S2. Distribution of maturing sockeye salmon caught between April and June for 1956-1970, adapted from French et al. (1976).

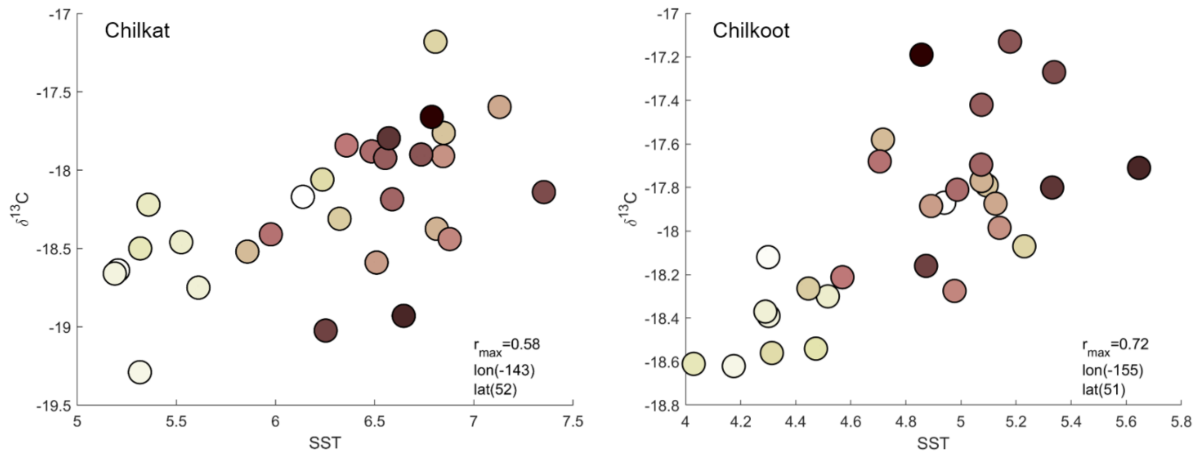

Figure S3. Scatter plot of the SST (averaged for Jan-Jun) vs $\delta^{13}C$ in maturing sockeye salmons from Chilkat and Chilkoot stocks in the SE Alaska. The dot color changes as a function of year, going from white (1968) to dark brown (1998).

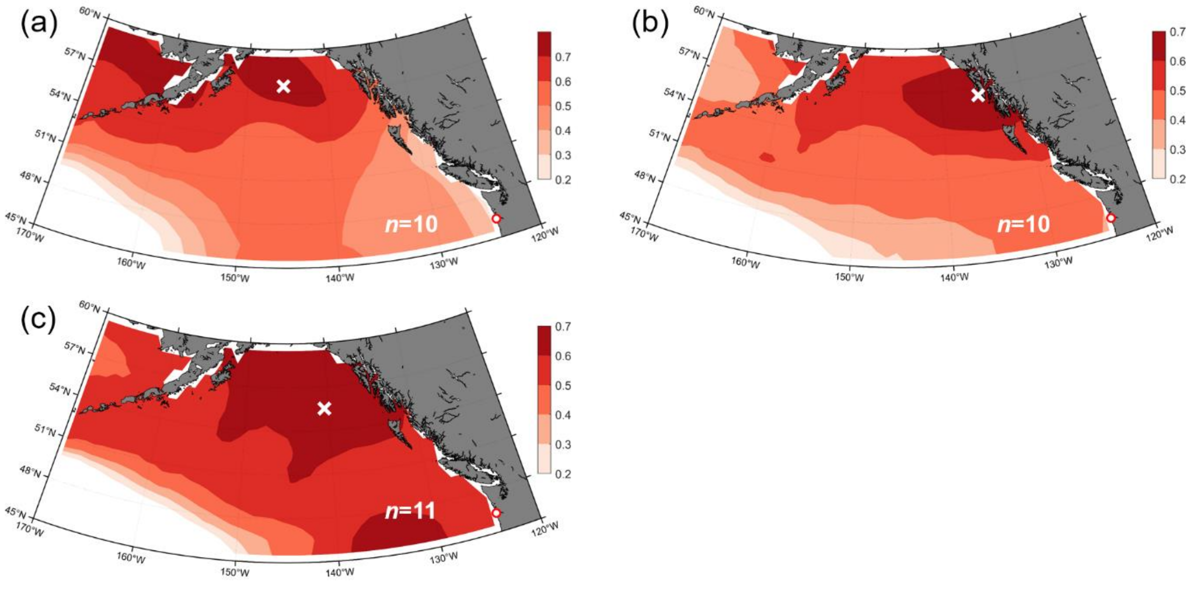

Figure S4. Estimated feeding grounds during the last year at sea of Okanagan sockeye salmon stock (Columbia) subsampling the time series every 3 years starting at (a) t_0, (b) t_0+1 and (c) t_0+2. Strongest correlation coefficient between SST and $\delta^{13}C$ values is indicated by a white cross. Locations of the point of entry in the freshwater system of the different stocks is shown (red circle). The number of samples *n* is shown.

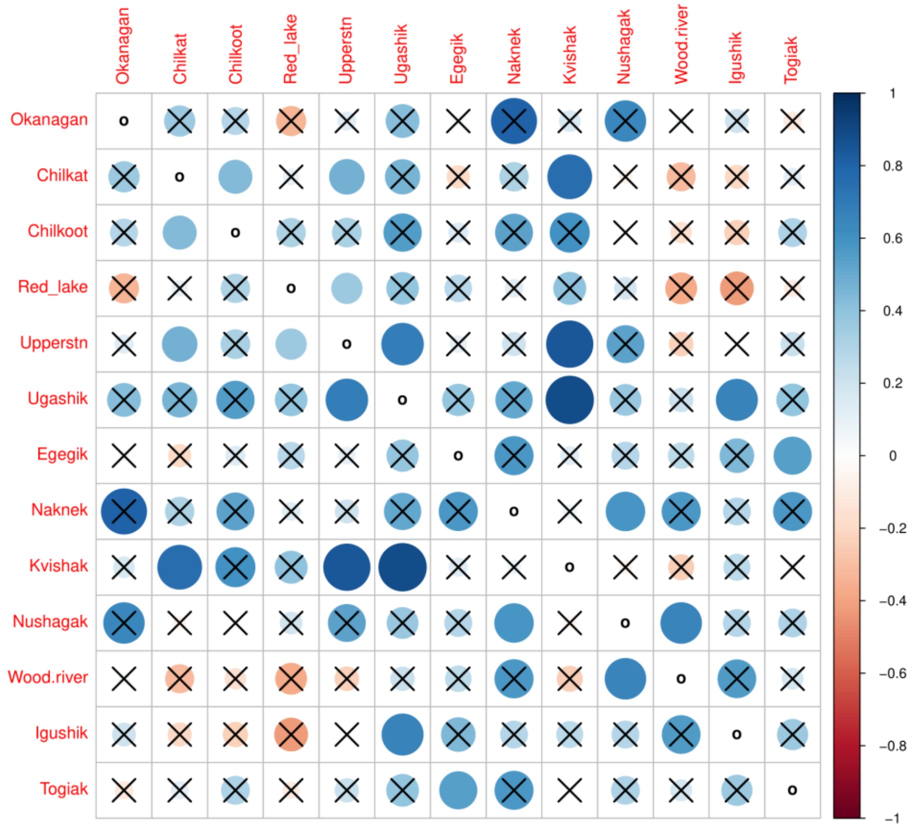

Figure S5. Correlation matrix of $\delta^{15}N$ values of several sockeye salmon stocks. Non-significant correlations are crossed out ($p > 0.05$).
